# 

**Published:** 1846-07

**Authors:** 


### CONTENTS OF Nº XLIII

OF THE

### British and Foreign Medical Review.

JULY, 1846.

---

### PART FIRST.—Analytical and Critical Reviews.

---

PAGE

ART. I.—Die Hauptformen der Seelenstörungen in ihren Beziehungen zur Heilkunde. Von Dr. MAXIMILIAN JACOBI . . . . . . 1

The principal Forms of Insanity in relation to Treatment. By Dr. MAXIMILIAN JACOBI.

ART. II.—Physiologie Pathologique, ou Recherches cliniques, expérimentales, et microscopiques sur l'Inflammation, la Tuberculization, les Tumeurs, la formation du Cal, &c. Par H. LEBERT, M.D. . . . . . 18

Pathological Physiology, or Clinical, Experimental, and Microscopical Researches on Inflammation, Tuberculization, Tumours, the formation of Callus, &c. By H. LEBERT, M.D.

ART. III.—Glanders and Farcy in the Horse. By WILLIAM PERCIVAL, M.R.C.S., Veterinary Surgeon of the 1st Life Guards, &c. . . . . 35

ART. IV.—Untersuchungen und Erfahrungen im Gebiete der Chirurgie. Von Dr. FRIEDRICH PAULI . . . . . . . . 48

Researches and Practical Observations in Surgery. By Dr. FRIEDRICH PAULI.

ART. V.—1. An Essay on the Use of Narcotics and other remedial agents, calculated to produce Sleep, in the Treatment of Insanity. By JOSEPH WILLIAMS, M.D. . . . . . . . . 55

2. Practical Notes on Insanity. By JOHN BURDETT STEWARD, M.D. . ib.

ART. VI.—Traité des Maladies des Articulations. Par A BONNET, Professeur de Clinique chirurgicale à l' Ecole de Médecine de Lyon, &c. . . 62

A Treatise on Diseases of the Joints. By A. BONNET, Professor of Clinical Surgery in the Medical School of Lyons, &c.

ART. VII.—Letters on Pulmonary Phthisis, delivered in Jervis-street Hospital; comprehending the Pathology, Diagnosis, and Treatment of the Disease, with an Appendix. By J. T. EVANS, M.D. . . . . . 85

§

4299

PAGE

Art. VIII.—Delle Alterazioni Patologiche delle Arterie per la legatura e la torsione, Esperienze ed Osservazioni di Luigi Porta, Professore di Clinica Chirurgica nell' I. R. Universita di Pavia. Con tredici Tavole in Rame . 89

Experiments and Observations on the Pathological Alterations of Arteries by Ligature and Torsion. By Luigi Porta, Professor of Clinical Surgery in the Royal University of Pavia.

Art. IX.—A Memoir on Amputation of the Thigh at the Hip-Joint (with a successful Case). By William Sands Cox, f.r.s. . . . 111

Art. X.—Sui Pregi e Doveri del Medico. Del Professore Roberto Sava . 115

On the Deserts and Duties of the Physician. By Professor Robert Sava.

Art. XI.—Scrofula; its Nature, its Causes, its Prevalence, and the Principles of Treatment. By Benjamin Phillips, f.r.s., Assistant Surgeon to the Westminster Hospital . . . . . . . 124

Art. XII.—Traité des Maladies du Sein, comprenant ses Affections simples et cancéreuses. Par J. Carpentier-Méricourt, m.d. . . . 156

A Treatise on Diseases of the Breast, both simple and cancerous. By J. Carpentier-Méricourt, m.d.

Art. XIII.—Lectures, illustrative of various Subjects in Pathology and Surgery. By Sir Benjamin Brodie, Bart., f.r.s. . . . . 160

Art. XIV.—The Structure and Functions of the Female Breast, as they relate to its Health, Derangement, and Disease. By E. W. Tuson, f.r.s., Surgeon to the Middlesex Hospital . . . . . 173

Art. XV.—Medical Notes on China. By John Wilson, m.d., f.r.s., f.s.s., Inspector of Naval Hospitals and Fleets . . . . 179

Art. XVI.—De Quantitate relativâ et absolutâ Acidi Carbonici, ab Homine sano et ægroto exhalati. Auctore Adolpho Hannover, Med. Lic., Med. Secund. nosocomii Regii Fredericiani, &c. &c. . . . . 189

On the relative and absolute Quantity of Carbonic Acid exhaled by Man in Health and Disease. By Adolphus Hannover, m.d., &c.

Art. XVII.—A Practical Treatise on Abdominal Hernia. By Thomas Pridgin Teale, f.l.s., Fellow of the Royal College of Surgeons, and Surgeon to the Leeds General Infirmary . . . . . 193

Art. XVIII.—The Young Stethoscopist; or, the Student's Aid to Auscultation. By H. J. Bowditch, m.d. . . . . . 209

Art. XIX.—Etudes de Géologie Médicale sur la Phthisie Pulmonaire et la Fièvre Typhoide, dans leurs Rapports avec les Localités Marécageuses, &c. Par S. Ch. Boudin, &c. . . . . . . , 211

Medico-Geological Studies on Pulmonary Phthisis and Typhoid Fever, in their Relations to Marshy Districts. By S. Ch. Boudin, &c.

Art. XX.—The Vital Statistics of Glasgow for 1843 and 1844. Drawn up, &c. &c., by Alexander Watt, ll.d., City Statist . . . . 215

Art. XXI.—Essai sur les Tumeurs Solides Intra-Thoraciques. Par J. M. H. Gintrac. Thèses de Paris, Janv. 1845 . . . . 216

Essay on Solid Intra-Thoracic Tumours. By J. M. H. Gintrac, &. &c.

PAGE

ART. XXII.—Die Nervenkraft im Sinne der Wissenschaft gegenüber dem Blutleben in der Natur. Rudiment einer naturgemässern Physiologie, Pathologie und Therapie des Nervensystems. Von Dr. CARL JOS. HEIDLER, &c. . 218

The Nervous Power of Science versus the Blood-life of Nature. A Fragment of a Natural Physiology, Pathology, &c., of the Nervous System. By Dr. C. J. HEIDLER, Imperial Austrian Councillor, Senior Official Physician to the Baths at Marienbad, &c.

ART. XXIII.—Liebig's Physiology applied in the Treatment of Functional Derangement and Organic Disease. With Observations upon Hahnemann's Practice.—Part I. The Heart, Lungs, Stomach, Glands, Joints, Bones, &c., with Cases, showing the Advantages of Modern Science over Former Methods in the Treatment of Disease. By JOHN LEESON, Practitioner of Medicine, Divisional Surgeon of the Metropolitan Police Force, &c. &c. . . 221

ART. XXIV.—On the Analysis of the Blood and Urine, in Health and Disease; and on the Treatment of Urinary Diseases. By G. OWEN REES, M.D., F.R.S., F.G.S., &c. Second Edition . . . . . 223

### PART SECOND.—Bibliographical Notices.

ART. I.—Memoir on the Field Carriage of Sick and Wounded Soldiers in the Bengal Army. By J. S. LOGIN, M.D., Surgeon to the British Residency at Lucknow, Superintendent of H. M. the King of Oude's Hospital, late Surgeon to the Commander-in-Chief in India, &c. &c. . . . . 225

ART. II.—Lettre sur la Syphilis ou Vues Nouvelles sur la Nature et le Traitement de la Maladie Vénérienne. Par F. S. RATIER . . . 226

New Views of the Nature and Treatment of Syphilis. By F. S. RATIER.

ART. III.—Collectanea Medico-Chirurgica; or, Cullings from the Case-Book of a General Practitioner in Medicine, Surgery, and Midwifery. By W. MACLURE, Esq., Surgeon, &c. &c. . . . . ib.

ART. IV.—An Atlas of Anatomical Plates of the Human Body, accompanied with Descriptions in Hindustani. By FRED. G. MOUATT, M.D., assisted by MOOUSHI NUSSEERUDIN AHMUD . . . . . 227

ART. V.—The Sanative Influence of Climate. By Sir JAMES CLARK, Bart., M.D., F.R.S., Physician in Ordinary to the Queen and to the Prince Albert. Fourth edition . . . . . . . 228

ART VI.—On the Temperature of the Earth and Sea, in reference to the Theory of Central Heat: a Lecture delivered at the Royal Institution. By ALFRED S. TAYLOR, F.R.S., &c. . . . . . . 229

ART. VII.—Phrenology—its Nature and Uses; an Address to the Students of Anderson's University, at the opening of Dr. Weir's first Course of Lectures on Phrenology in that Institution, Jan. 7, 1846. By ANDREW COMBE, M.D. . 230

PAGE

ART. VIII.—1. Outlines of the Arteries, with short Descriptions. By JOHN NEILL, A.M., M.D. . . . . . . . 231

2. Outlines of the Nerves, with short Descriptions. By JOHN NEILL, A.M., M.D., Demonstrator of Anatomy in the University of Pennsylvania, &c. . ib.

ART. IX.—Percussion und Auscultation des Herzens im gesunden und kranken Zustande. By LIBERAL GUNZBURG, M.D. . . . . . 232

A Treatise on Percussion and Auscultation of the Heart in the Healthy and Diseased States. By LIBERAL GUNZBURG, M.D.

ART. X.—Urologie. Traité des Angusties ou Rétrécissements de l'Urètre, et de leur traitement rationnel. Par le Dr. LEROY-d'ETIOLLES . . 233

Urology. Treatise on Strictures of the Urethra, and their rational treatment. By Dr. LEROY-D'ETIOLLES.

ART. XI.—Some Observations on Organic Alterations of the Heart, and particularly on the beneficial employment of Iron in the treatment of such cases. By S. SCOTT ALISON, M.D., M.R.P.L., and Physician to the Northern Dispensary . 234

---

### PART THIRD.—Natural History and Treatment of Diseases.

I.—On the recent Progress and future Prospects of Practical Medicine. By ELISHA BARTLETT, M.D., Professor of Medicine in the University of Maryland . . . . . . 235

II.—On the Natural History and Simple Treatment of Wounds. By ISAAC GILCHRIST, M.D. . . . . . . 238

IV.—Extracts from Correspondence . . . . . 243

---

### PART FOURTH.—Original Reports and Memoirs.

Report on the Progress of Human Anatomy and Physiology in the years 1844-5. By JAMES PAGET, Lecturer on General and Morbid Anatomy and Physiology, and Warden of the Collegiate Establishment, at St. Bartholomew's Hospital. Part II . . . . . 261

---

Books received for Review . . . . . . . 292

